# Dropout and completion in iCBT for university students: Insights from a thematic analysis

**DOI:** 10.1016/j.invent.2025.100831

**Published:** 2025-05-14

**Authors:** Jurrijn A. Koelen, Lisa de Koning, Matilda K. Nottage, Anke M. Klein, Claudia M. van der Heijde, Peter Vonk, Reinout W. Wiers

**Affiliations:** a Addiction, Development, and Psychopathology Lab, Department of Psychology, University of Amsterdam, Nieuwe Achtergracht 129, 1001NK Amsterdam, the Netherlands; b Department of Psychology, Health and Technology, University of Twente, P.O. Box 217, 7500 AE Enschede, the Netherlands; c Department of Research, Development and Prevention, Student Health Service, University of Amsterdam, Oude Turfmarkt 151, 1012 GC Amsterdam, the Netherlands; d Center for Urban Mental Health, Oude Turfmarkt 145-147, 1012 GC Amsterdam, the Netherlands

**Keywords:** Internet-based cognitive behavioral therapy, Depression, Anxiety, Dropout, University students, Adherence

## Abstract

Online cognitive behavioral therapy (iCBT) is a promising treatment for depression and anxiety among university students but faces high dropout rates. Understanding the reasons behind dropout or completion can help improve the implementation of iCBT in educational settings. Semi-structured phone interviews were conducted with 32 students who dropped out early (*n* = 9), midway (*n* = 12), or completed (*n* = 11) guided or unguided iCBT in the context of a randomized controlled trial. Data were analyzed using Braun and Clarke's (2012) thematic analysis. Common themes among dropouts included personal factors (like competing priorities), perceived difficulty or redundancy of the intervention, and lack of human interaction. Early dropouts uniquely cited disbelief in the intervention's efficacy and preference for other mental health support. Midway dropouts mentioned issues with the interactivity, feedback, content, perceived effectiveness, and lack of personalization. Completers had positive initial impressions, valued the online format, found the exercises and guidance helpful, and felt cared for. The themes identified among participants who dropped out from or completed the iCBT intervention provide valuable insights into factors which may be of importance for retention. Implications regarding setting expectations, participant selection, interactive functionalities, personalized feedback, and the role of therapist guidance are discussed.

## Highlights

• Reasons for student dropout from iCBT differ at various stages of the intervention
• Early dropouts from iCBT among university students were often due to disbelief in the intervention's efficacy.
• Midway dropouts highlighted issues related to the intervention's design.
• Over 40% of participants who did not complete the intervention reported no longer needing support.
• Participants who completed the iCBT intervention appreciated the online format and the therapist guidance.

## 1 Introduction

Mental health issues among university students are a pressing public health concern with significant implications for their well-being and academic success (Alonso et al., 2018). A global survey conducted by the World Health Organization revealed that 31 % of university students meet the criteria for one or more mentahealth disorders, with depression and anxiety being the most frequently reported (Auerbach et al., 2019). These conditions often arise from the complex challenges faced during young adulthood, including personal and social development, increased independence, and heightened responsibilities. Additionally, academic pressures and financial strains contribute significantly to the mental health burden of students (Karyotaki et al., 2020). This interplay between personal and academic stressors can create a vicious cycle, where mental health issues exacerbate difficulties in managing everyday challenges, thereby worsening the symptoms further. Research highlights that mental distress can negatively impact academic performance, underscoring the importance of addressing these issues effectively (Karyotaki et al., 2020; Bolinski et al., 2020). Consequently, developing and implementing effective treatments that are readily accessible is crucial.

Internet-based cognitive behavioral therapy (iCBT) has emerged as a promising approach for treating and preventing common mental health issues. Built on the principles of cognitive behavioral therapy, iCBT is delivered online and can be offered with varying degrees and types of guidance (Koelen et al., 2022). Evidence supports the efficacy of iCBT in treating a range of mental health concerns, including anxiety and mood disorders, also among students (Domhardt et al., 2019; Harrer et al., 2019; Karyotaki et al., 2021). Compared to traditional face-to-face therapy, iCBT offers significant advantages in terms of accessibility, availability, and potential cost-effectiveness for both patients and healthcare providers. The online format also provides benefits related to privacy, confidentiality, and anonymity, which can help reduce the stigma often associated with seeking mental health care (Knowles et al., 2015; Gericke et al., 2021).

Despite its advantages, iCBT faces a notable challenge: high dropout rates. Research indicates that treatment dropout is a significant issue, as early termination diminishes the likelihood of intervention benefits and complicates the assessment of the intervention's effectiveness (Fernandez et al., 2015; Treanor et al., 2021). Adherence to iCBT programs is typically lower compared to face-to-face CBT, with systematic reviews showing that approximately one-third to half of participants drop out before completing the program (Koelen et al., 2022; Treanor et al., 2021).

### 1.1 Association between adherence and outcome in iCBT

The relationship between adherence rates and treatment outcomes is complex. Adherence is commonly considered a proxy for engagement and exposure to therapeutic content, based on the assumption that higher adherence leads to improved outcomes. Yet some individuals discontinue therapy after early improvement or spontaneous remission, suggesting that not all dropouts reflect failure (Reich and Berman, 2020). Likewise, iCBT trials have found similar outcomes across varying adherence levels (Koelen et al., 2024), and some participants benefit significantly despite only partially completing the intervention (Christensen et al., 2009; Karyotaki et al., 2017). This suggests that additional factors may influence this relationship, such as depth of interaction with therapeutic principles (Donkin et al., 2011) or individual differences in dosage needs (Eysenbach, 2005). In light of these contradictory findings, adherence remains a critical focus for internet interventions, as it increases the likelihood of participants engaging with core therapeutic components. Adherence is often associated with engagement behaviors, which are essential for the success of behavior change interventions (Donkin et al., 2011; Kelders et al., 2012). These considerations highlight the need for further research to disentangle the multidimensional nature of adherence and its relationship with therapeutic outcomes. Few studies have examined factors associated with adherence at different stages of the intervention.

### 1.2 Factors associated with dropout

Existing reviews often focus on quantifiable factors such as demographics and clinical characteristics, which have yielded mixed results regarding their impact on dropout rates (Christensen et al., 2009; Rost et al., 2017; Treanor et al., 2021). However, qualitative research exploring the experiences of individuals undergoing iCBT is relatively scarce. Such studies could provide deeper insights into the motivations and barriers faced by participants. One critical factor identified in previous research is the role of human or therapist support. Evidence suggests that personalized support can enhance the perceived helpfulness of the intervention, while the absence of personal contact and delays in receiving therapist responses may negatively affect engagement (Gerhards et al., 2011; Lillevoll et al., 2013; Knowles et al., 2015; Rozental et al., 2015; Holst et al., 2017; Fernández-Álvarez et al., 2017; Pedersen et al., 2020). Conversely, some participants have reported positive experiences with online therapeutic relationships, highlighting the potential for effective engagement even in the absence of face-to-face interactions (Beattie et al., 2009).

Research specifically focusing on students' experiences with iCBT is limited. Given the unique context of university life, including high digital literacy and busy schedules, students may have distinct needs and challenges that warrant separate investigation. Evidence also suggests that younger age is associated with a higher risk of dropout, emphasizing the need to understand dropout factors in young adult populations (Edmonds et al., 2018; Karyotaki et al., 2018). A recent study involving 29 university students who did not complete an iCBT program (the transdiagnostic iCare Prevent program) found that common reasons for dropout included time constraints and mismatched needs between the intervention and participants' expectations. Intervention-related barriers included a preference for more personal contact and lack of pressure to complete subsequent modules (Ciharova et al., 2023). However, this study only addressed students who did not complete the program in its entirety.

### 1.3 Aim of the present study

Exploring the experiences of students who drop out at various stages of an iCBT program, as well as those who complete it, is essential for improving retention rates and potentially optimizing iCBT interventions. The current study aims to deepen our understanding of factors influencing iCBT dropout and completion of iCare Prevent program among university students. By examining three stages of adherence—early dropout, midway dropout, and completion—this study seeks to uncover how initial impressions, and the content of the intervention affect dropout rates and whether reasons for dropping out differ at various stages of the program. This investigation will provide valuable insights into improving the design and implementation of iCBT interventions tailored for student populations.

## 2 Methods

### 2.1 Design of the original trial

This study is part of a three-armed randomized controlled trial (RCT) conducted at a Dutch university. The RCT compared two versions of an internet-based cognitive behavioral therapy (iCBT) intervention (the transdiagnostic iCare Prevent program): one with human guidance (HG) and one with computerized guidance (CG), against a control group receiving care as usual (CAU). Participants were eligible for the RCT if they met the following criteria: (1) aged 16 or older, and (2) exhibited mild to severe symptoms of depression, and/or anxiety measured through our eSurvey (Koelen et al., 2024). Exclusion criteria were: (1) comorbid bipolar disorder or psychotic disorders, according to the Mini International Neuropsychiatric Interview (M.I.N.I.; Sheehan et al., 1998); (2) active high suicide risk; (3) current psychological treatment for depression or anxiety; (4) inadequate internet access; and (5) lack of written informed consent.

### 2.2 Participants and procedures

The study was conducted within a university-wide research project. Students enrolled at the University of Amsterdam were invited (in separate cohorts) via email through the university's research platform (LOTUS). When unresponsive, a maximum of two reminder emails were sent (1 and 2 weeks after the first invitation). Students were further informed about the study through study advisors and counsellors who were asked to refer potentially eligible or interested students. For the original RCT, we recruited 412 participants, with 274 assigned to one of the two iCBT intervention groups. Dropout rates were notable, with 28 % (77 participants) dropping out early (i.e., before completing the first module), 26 % (72 participants) dropping out midway (after 3–5 modules), and 21 % (58 participants) completing the full program (7 or 8 modules).

**Fig. 1.**
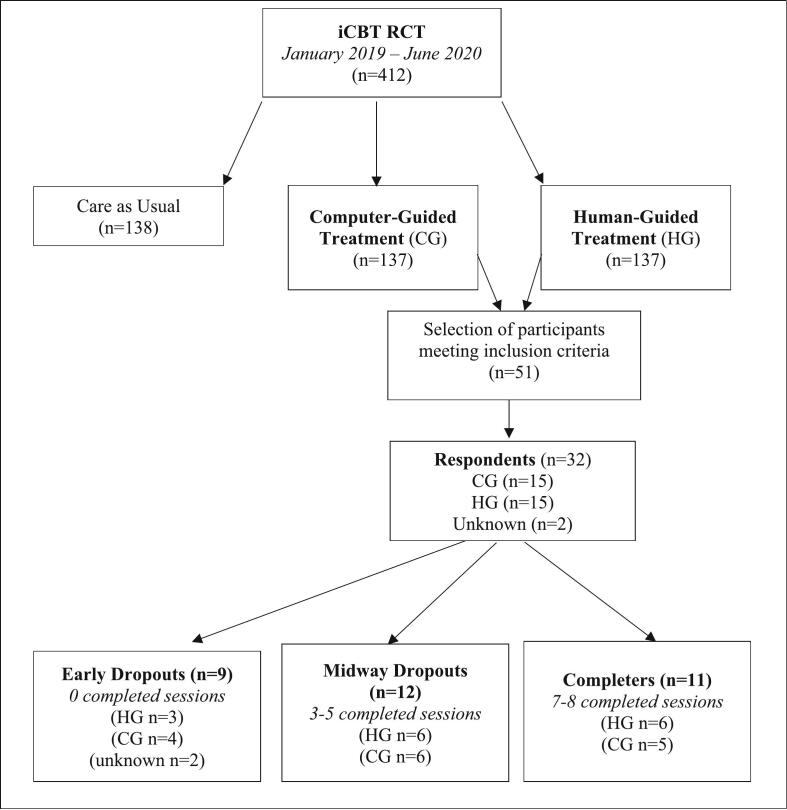
Flow chart.

For the current qualitative study, we selected 51 students (Bachelor's and Master's) who had stopped using the intervention for at least two months. Participants were selected using a pragmatic recruitment strategy. To enhance recall accuracy and maximize response rates, we prioritized inviting more recent dropouts from the intervention. Students were contacted via email with an invitation to participate in a phone interview, and 32 accepted the invitation (63 %) across both the CG and HG conditions (Fig. 1). This group included 9 early dropouts, 12 midway dropouts, and 11 completers. Demographic and clinical characteristics of these participants are detailed in Table 1. The age range was 18 to 40 years (M = 23.5; SD = 4.5), with 66 % identifying as female (*n* = 21). Baseline anxiety, measured with GAD-7 (Spitzer et al., 2006) (M = 9.1; SD = 4.7; *n* = 30), and depression, measured with PHQ-9 (Kroenke et al., 2001) (M = 10.8; SD = 5.4; n = 30), did not significantly differ from the remaining RCT sample [t(409) = 0.00, *p* > 0.99), and t(409) = 0.10, *p* = 0.92), respectively]. Statistical comparisons showed no significant differences in age [F(2, 27) = 0.70, *p* = 0.51], depression [F(2, 27) = 1.75, *p* = 0.19], or anxiety [F(2, 27) = 0.44, *p* = 0.65] between the three groups. Gender distribution across groups was even [X^2^(4, *N* = 32) = 8.37, *p* = 0.08], as was the distribution between HG and CG conditions [X^2^(4, N = 32) = 5.67, *p* = 0.23]. The study specifically targeted Bachelor and Master students, excluding PhD students.

**Table 1:** Demographic and clinical characteristics of the 32 interviewed participants.

ID	Group	Condition	Gender	Age (yrs)	PHQ-9	GAD-7	PD	CD
#1	EARLY	CG	Male	23	8	4	None	
#2	EARLY	CG	Female	25	15	14	None	
#3	EARLY	CG	Male	20	8	4	Depression	
#4	EARLY	CG	Male	24	19	17	Depression	Hypomanic episode (lifetime)
#5	EARLY	HG	Male	26	12	7	Depression	Agoraphobia
#6	EARLY	HG	Female	29	22	12	Depression	
#7	EARLY	HG	Female	19	13	16	Depression	Social phobia
#8	EARLY	n.a.	n.a.	n.a.	n.a.	n.a.	n.a.	
#9	EARLY	n.a.	n.a.	n.a.	n.a.	n.a.	n.a.	

Mean (SD) early (*N* = 9)				23.7 (3.5)	13.9 (5.3)	10.6 (5.5)		
#10	MID	CG	Male	20	17	14	Depression	Social phobia
#11	MID	CG	Female	20	16	14	Depression	Panic disorder (lifetime)
#12	MID	CG	Female	20	16	12	Depression	Generalized anxiety disorder
#13	MID	CG	Female	31	7	5	Mixed anxiety-depression	
#14	MID	CG	Male	24	4	10	Social phobia	Agoraphobia
#15	MID	CG	Male	24	4	3	None	
#16	MID	HG	Male	19	15	8	Depression	Social phobia
#17	MID	HG	Female	24	4	8	GAD	Panic disorder (lifetime)
#18	MID	HG	Female	18	18	14	Depression	GAD
#19	MID	HG	Male	25	10	5	Social phobia	Panic disorder (lifetime)
#20	MID	HG	Female	25	7	4	None	
#21	MID	HG	Female	18	8	9	Social phobia	Agoraphobia
Mean (SD) MID (N = 12)				22.3 (3.8)	10.5 (5.5)	8.8 (4.0)		
#22	COMPLETE	CG	Female	20	6	2	None	
#23	COMPLETE	CG	Female	22	9	11	GAD	
#24	COMPLETE	CG	Male	25	10	6	Depression	OCS
#25	COMPLETE	CG	Female	25	10	9	None	
#26	COMPLETE	CG	Female	22	7	11	Depression	Social phobia
#27	COMPLETE	HG	Female	26	8	10	Social phobia	
#28	COMPLETE	HG	Female	21	20	9	Depression	
#29	COMPLETE	HG	Female	27	5	3	Mixed anxiety-depression	
#30	COMPLETE	HG	Female	20	16	21	Depression	OCS
#31	COMPLETE	HG	Female	40	5	6	Panic disorder	Hypomanic episode (lifetime)
#32	COMPLETE	HG	Male	22	5	5	None	
Mean (SD) COMPLETE (N = 11)				24.5 (5.7)	9.2 (4.8)	8.5 (5.2)		
Mean (SD) total sample (N = 32)				23.5 (4.5)	10.8 (5.4)	9.1 (4.7)		

*Note.* PHQ = Patient Health Questionnaire; GAD = Generalized Anxiety Disorder; PD = primary diagnosis; CD = comorbid diagnosis; OCS = obsessive-compulsive disorder.

### 2.3 Interventions

Both iCBT interventions were composed of 8 modules but differed in the type of guidance provided (Koelen et al., 2024). Participants in the HG condition received personalized feedback from an ‘e-coach’ (a healthcare psychologist or psychology master's student) involving a tailored response with encouragement and tips, within 5 working days after each module. Participants in the CG condition also received some feedback, but only in the form of automated responses. These brief messages offered a standardized summary of the module content, validation of progress, and motivating cues. Both interventions were sequential, requiring participants to complete core modules in a specific order. In modules five and six, participants chose whether to focus on symptoms of depression (problem-solving) or anxiety (exposure in daily life). Additionally, optional modules addressing transdiagnostic factors such as self-worth, alcohol use, and rumination were available in modules 2 through 7 (Koelen et al., 2024). Adherence (in both conditions) was supported through several measures: an integrated messaging function for technical and content-related questions, standardized email reminders (up to three per week) in case of inactivity, and monitoring for sharp increases in depressive symptoms with phone contact for suspected suicidal risk (Koelen et al., 2024). The main results of the trial indicated that there was a significant difference in adherence between the CG and the HG groups, in that the HG group completed more modules on average [(3.3 (SD = 3.0) vs. 2.5 (SD = 2.8)]. The completion rate for the HG condition was also significantly higher than that for the CG condition: 26.9 % versus 15.5 % (Koelen et al., 2024). Nonetheless, to optimize sample size, and because evidence suggests that there are no clear differences between clinician-guided and self-guided iCBT interventions in terms of effectiveness (Karyotaki et al., 2021; Lungu et al., 2022; Koelen et al., 2024), these groups were combined in the present study.

### 2.4 Data collection

Interviews were conducted between June 29, 2020, and October 30, 2020. Participants could choose to be interviewed in English (*n* = 19) or Dutch (*n* = 13). To ensure objectivity, interviews were conducted by a different researcher from those who had previously conducted their initial screening or, for the HG group, had provided their intervention guidance. Semi-structured interviews were designed to gather detailed feedback on participants' experiences with the iCBT intervention. The interview protocol, developed by the research team, included open-ended questions covering reasons for joining, initial expectations, overall experience with the training, reasons for dropping out (if applicable), opinions about the project, and suggestions for improvement. For those in the HG group, additional questions were included about the feedback received. Interviews ranged from 7 to 30 min, with an average length of 16.5 min. All interviews were recorded and transcribed by research assistants, with Dutch transcripts translated into English using DeepL and checked for accuracy by a researcher (LK). See Appendix A for the interview protocol.

### 2.5 Analysis

Quantitative data were analyzed using SPSS version 28.0.1.0 (IBM SPSS Statistics for Macintosh, 2020) to compare age, depression, and anxiety levels between the selected sample and the remaining RCT participants. Independent *t*-tests were used for comparisons of continuous variables, and ANOVA was employed to assess differences between the three groups. Chi-square tests analyzed the distribution of gender and treatment conditions across the groups. For qualitative analysis, Braun and Clarke's thematic analysis (TA) (Braun and Clarke, 2012) was employed, following a six-step process: (1) familiarization with the data through repeated reading of transcripts, (2) generating initial codes, (3) identifying and (4) reviewing ‘themes’ (key patterns emerging from the codes), (5) defining these themes, and (6) reporting the results. The analysis was conducted in three phases: one for early dropouts, one for midway dropouts, and one for completers. Data were initially coded independently by co-authors MN and LK using an online tool designed to facilitate Qualitative Content Analysis, i.e., QCAmap (Mayring, 2022). The final themes were developed through collaborative discussion. Combining the TA method with the QCA tool leverages QCA's replicability and TA's interpretative depth, thus enhancing the depth and rigor of qualitative data analysis.

## 3 Results

Interviewees provided detailed accounts of their subjective experience with the iCBT intervention and their motives for continuing or discontinuing their participation. First, we describe the overlapping themes mentioned across participant groups; subsequently, we summarize group-specific themes.

### 3.1 Dropout groups

#### 3.1.1 Overlapping factors associated with early and midway dropout

Interviews with the nine participants from the early dropout group revealed seven key themes, some of which could be further divided into subthemes (Table 2), and 10 key themes among the 11 participants from the midway dropout group. Considerable overlap in these groups related to: (1) personal factors (e.g., other priorities), (2) difficulty with the exercises, (3) redundancy of the intervention (e.g., finding support elsewhere), and (4) lack of human support.

**Table 2:** Themes and subthemes identified across the three conditions: early dropout, midway dropout, and completers.

Early dropout group (N = 9)		Midway dropout group (N = 12)		Completers group (*N* = 11)	
Themes and subthemes	*N*	Themes and subthemes	*N*	Themes and subthemes	*N*
**Personal factors**	**5**	**Personal factors**	**10**	**Personal factors**	**7**
Having other priorities	3	Lacking time	8	Having discipline/Grit	5
Forgetting	3	Change in circumstances	5	Feeling open to trying	4
Lacking grit	2	Forgetting	2	Having only mild symptoms	4
Change in Circumstances	1	Lacking grit	2	Having external encouragement	2
				Having an academic interest	1
**Difficulty of the intervention**	**6**	**Difficulty of the intervention**	**8**	**Pleasantness of the intervention**	**6**
Finding the intervention too difficult	4	Feeling it was a chore	5		
Feeling it was a chore	4	Finding it emotionally difficult	4		
		Finding it too time consuming	3		
		Finding the intervention intellectually difficult	2		
**Issues with the interactivity**	**2**	**Issues with the interactivity**	**8**	**Satisfaction with the interactivity**	**7**
Technical issues	1	Inadequate reminders	6	Online format	4
Asynchronous feedback	1	Poor intervention flow	5	Ability to choose their own pace	4
Lack of interactive elements	1	Technical issues	1	Reminders to complete modules	3
Easy to ignore assignments	1			Accessibility	2
				Seeing progress between modules	2
				Starting with a phone interview	1
**Issues with human interaction**	**6**	**Issues with human interaction**	**8**	**Helpful therapist guidance**	**6**
				Receiving guidance	5
				Knowing someone is there	4
				Feeling the guidance is tailored	3
					
		**Issues with the feedback and guidance**	**5**		
**Redundancy of the intervention**	**3**	**Redundancy of the intervention**	**6**	**–**	**–**
Receiving other treatment	3	Feeling better	3		
Not needing intervention	2	Receiving other treatment	3		
**Lack of belief in the efficacy of the intervention**	**8**	**–**	**–**	**Positive preconceptions of the intervention**	**7**
Expecting the intervention won't be tailored to their issues	6				
Expecting it won't help with severe symptoms	4				
				**Positive first impressions**	**5**
**–**	**–**	**Issues with the content and/or exercises**	**5**	**Satisfaction with the content and/or exercises**	**10**
				Enjoyed the exercises	10
				Enjoyed the psychoeducation	7
				Enjoyed the optional modules	5
					
**–**	**–**	**Unhelpfulness of the intervention**	**4**	**Effectiveness of the intervention**	**11**
				Helpful	5
				Gained insights into their mental health	5
				Learnt to understand and address problems	5
**Preference for other mental health support**	**6**	**Preference for other mental health support**	**2**	**–**	**–**
Preferring a different format	4				
Preferring a different therapeutic approach	2				
**–**	–	**Not specific to the participants' issues**	**5**	**–**	**–**
		Addressed issues irrelevant to the participant	4		
		Not personalized	3		
		Not suited for severe symptoms	2		

*Note. N* refers not to the number of individual participants who mentioned the code at least once.

Personal factors were more prevalent in the midway dropout group yet overlapped considerably with the experiences in the early dropout group. Notably, participants in both groups mentioned forgetting about the program, lacking motivation, and changes in personal circumstances as factors contributing to dropout. For example, one participant mentioned: *“Yeah, cause uh, I think that people who have like anxiety and depression and things like that usually have a problem with motivation, and that motivating yourself to do something like that is quite difficult for them, and that having a person to tell them ‘you can do this, you can do this’ is more motivating than having a text that tells you you can do it.”* The COVID pandemic was mentioned as a major change in personal circumstances: *“Uhm, I moved- I left Amsterdam for two months and I went to stay with my mom. So I yeah, everything was quite different.”* And: *“Honestly I thought it was pretty good. The one thing that I would say was like, it was pretty like… I kept forgetting to, like, do the sessions and I never ended up doing them and then eventually when corona happened I lost the motivation. But I think that was more an external thing than the uh, actual programme itself.”*

Second, over 60 % of the participants in both groups found the intervention difficult. They experienced the exercises either as a “chore” or found them (emotionally or intellectually) difficult. One participant remarked: “*It required a lot of processing, as if I was doing homework. Now like, I'm going to try for some help, and it's like an extra burden*.” Another expressed: *“Yeah, yeah, maybe uh an improvement could be to… say less things, and – because we had to answer some questions that were based on a lot of background, I'd say, and I felt like it was too much and it took me way too much time to complete the questions.”* One participant mentioned emotional difficulties: “*I think it was really really stressful, and I would say scary”*.

Third, roughly 40 % of the participants in both groups found support elsewhere, felt better or did not need the intervention due to other reasons. Finally, over 60 % of participants in both groups missed human interaction. As expressed by one of the participants: *“I think that if, maybe if there was like uh introductory… more of an introductory conversation with uhm, the student and the mental health person, maybe then it would feel like a bit more personal because then they would know more about you as a person, rather than just like reading your responses to the forms and things.”* Another response about this lack of interaction was as follows: *“It doesn't even have to be someone like uh a mental health professional or somebody, but maybe even like someone else who signed up for the programme. That uh, would be possibly linked into my group, or that had similar struggles or something. Maybe then that would motivate me more, because we could also help each other a little maybe, so it would be more, like, interactive, the experience”*. And: “*Mh... uh, it felt kind of like you don't have that much human contact, because I feel like in this- in cases like this, you kind of need some motivation from a human to do that, and since it was just in the platform it kind of felt dehumanizing… yeah… basically more human interaction would be cool.”* Some participants mentioned the option of peer support. Only two participants (one in each group) attributed their dropping out to technical difficulties.

#### 3.1.2 Specific factors associated with early dropout

Two factors characterized the early dropout group: (1) lack of belief in the intervention beforehand, and (2) preference for other mental health support (see Table 2). A large proportion of participants in this group (*n* = 6) expected that this intervention would not be tailored to their needs: *“It didn't align with me, so to speak. It was uh, yes… the problems they were talking about there, those just weren't the case for me.”* Four participants inferred from the first session that the training was best suited to mild mental health issues, whereas they felt that their issues were more severe.

Secondly, six participants expressed a preference for another type of mental health support; either a different format or a different therapeutic approach: “*it feels legit and it feels scientific and uh, if I do it I do feel like I will be helped in some kind of way but I don't know how. But then that's not really, as I said, it's not the right format for me. I believe in it, but, not the right format. It's not motivating for me to do it.”* Two preferred a different therapeutic approach. For example: *“And then I thought that what I talked to her about didn't quite connect, kind of, to the program that was offered on the website. And then I thought maybe this is not the way I want to approach it.”*

#### 3.1.3 Specific factors associated with midway dropout

Five specific factors characterized the interviews of the midway dropouts: (1) issues pertaining to the lack of interactivity, (2) issues with feedback and guidance (e.g., too superficial), (3) issues with the intervention and the exercises (e.g., too simplistic), (4) a lack of perceived effectiveness, and (5) a lack of personalization (e.g., not specific to their issues).

First, most participants (*n* = 8) from the midway dropout group reported that the interactivity of the intervention was not satisfactory. Some specified that they would have liked additional reminders to complete individual exercises, e.g.: “*I think, that my expectation was to get a little bit more, to get an email from that daily, for example: ‘Oh here's your breathing exercise for today and don't forget to fill out your journal’ or something. […] that would have helped too*”. Another said: *“And maybe there's- maybe if there were more reminders or something like that. But there were email reminders that I didn't just see.”* The second subtheme concerned the intervention's flow. Five participants recalled that the intervention lacked structure or featured long pieces of text and a complex design, which led them to drop out.

Second, about half of the participants in the midway dropout group (*n* = 5) mentioned factors related to feedback and guidance. They disclosed that the feedback they received after each module was not helpful, superficial, and they felt it had no additional effect: “*Uhm, but that wasn't actually the case in my experience. It was more like a kind of, yes, it felt like a kind of, uh, coach who made sure you did the training every time. And that was good too. I see you completed this little exercise, good on you, and I see you completed this little exercise, good on you. And I see you didn't fill this one out very extensively, why is that? Uhm, can you do that next time? Uhm, that remained very superficial as far as I was concerned on the, on ‘how did the online session go’ instead of ‘what did you fill out substantively in that session, and how can I as an associated psychologist offer you some help in addition to the online training’. And that was kind of unfortunate.”*

Thirdly, five participants expressed issues with the content of the intervention, as they felt that the intervention was simplistic, not stimulating enough and that the focus was too much on self-reflection, without practical implications*.* For example: *“Um, to be honest, from the very beginning from my interview, um, they kind of told me that I should also seek therapy, and that this would not necessarily be enough for me. So I always kept that in mind, and I think that was right because it was definitely useful on some- with some aspects, but I had a bit more complex issues to deal with and a lot of trauma to go through, so then it felt a bit simplistic at times.”*

Four participants felt the intervention wasn't helpful to them. For example: *“But on the whole I can't say I feel like it was very impactful, but Corona didn't help with that I think.”*

Finally, five participants felt that the intervention did not specifically enough address issues that they were struggling with, as one participant noted: “*Yes, exactly, and with me the cause was simply not study-related, but some other personal things. […] where it just wasn't quite adequate enough for me, but I think just the normal student in that regard who just experiences a lot of stress these days, fear of failure, performance pressure, that they could be helped a lot*.” Another commented that: *“online therapy is difficult to structure so that it is directed towards the individuals and so that people feel like it is personalized and that it addresses their specific needs. So, I think that's just a limitation of online therapy in general sometimes, with sessions like these. But I also think it may be related to the fact that I have been… I've been doing therapy in person for quite a while, so that I'm used to a different format.”*

### 3.2 Completers

#### 3.2.1 Factors associated with completion

Interviews with the 11 participants from the completer group revealed eight key themes, with some themes consisting of various subthemes (see Table 2). Interestingly, most, yet not all, key themes brought up by completers positively paralleled the negative themes brought up in the dropout groups. Personal factors (having grit, being open to try), the content of the intervention, and therapist guidance contributed to completion. A positive first impression stood out as a unique factor for completers.

More than half of the completers (*n* = 7) attributed the success of their completion to the satisfaction with the intervention's design (e.g., doing it at their own pace, it being online): “*Uhm, it was very nice that I could do a lot of things in my own time. That if something didn't work out, like if I had planned to do it during a certain day but I had something else come up that I could always just do it the next day, there was no scheduled time, and so I think that's definitely a benefit of the, uhm, self-help aspect.”* Another respondent said: *“Um. I liked that each session started in the same way, that it asked me the same questions so I could actually see myself like changing my own behaviour and perspective, I liked that.”*

More than half (*n* = 6) found the guidance helpful. For example, one completer noted: *“Yes, and then with the personal… that there is also someone behind it who sort of, really watches. I've often had messages saying: you haven't finished your work, can you finish it? I thought, oh yes. So that you then don't slack off.”* And: *“I felt like I got some attention, um, and that somebody actually cared.”* Another remarked: *“And I also really enjoyed talking to the psychologist who was assigned to me. I don't remember her name, but she was very very kind and very supportive and very attentive, so, she was great, yeah.”*

Most completers (n = 7) expressed a positive preconception of the project and the RCT that the participants were part of. Five participants mentioned positive first impressions of the project and intervention which attributed to their continuation*: “I found the uhm... the first impression was actually positive. I thought it was very clearly explained and also uhm... uh let's say, there was no finger pointing or anything, more ‘this works so and so thus it's logical that you act so and so, but it can also be so and so’. So, in a clear way, without them saying that you must do this differently.”*

Ten participants enjoyed the exercises, and all experienced the intervention as effective. For example: “*All the reading and telling me about my how the psyche works a little bit, and these kind of tricks you can apply and this and this you can try. Uhm, just in terms of the knowledge I got out of it I found it extremely enjoyable.”* And: *“Um, but I did find it useful this plan, I don't remember the name, but choosing my problem and then step by step finding a solution and then predicting what could prevent me from not doing that and what can I do about that, like, I found that really really useful.”* Also: “*Yeah, yeah, I think it was good to… I think especially the thinking exercises were you had to list certain things like activities that you should do, I think those were… like, activities that made you active were the best ones for me.”*

## 4 Discussion

This qualitative study among 32 university students sought to identify factors influencing dropout and completion of an 8-module iCBT intervention aimed at reducing anxiety and depression among university students. Key themes emerged from interviews with early dropouts, midway dropouts, and completers, including personal factors, intervention difficulty, design, interaction quality, and personalized care. Different internal factors (e.g., individual motivation and mental health status) and external factors (e.g., perceived workload and support availability) impacted dropout and completion at various stages. The findings of this study underscore the importance of viewing adherence and dropout as multidimensional phenomena that must be interpreted in context. For instance, over 40 % (9/21) of participants who did not complete the intervention reported feeling better, no longer needing support, or having found help elsewhere. In what follows, we discuss five themes relevant to improving iCBT implementation: expectations, participant selection, interactive functionalities, personalized care, and human guidance.

### 4.1 Expectations

Student feedback highlighted that dropout reasons were significantly influenced by initial expectations and impressions. Early dropouts, who did not complete any modules, based their decisions on perceived efficacy and trust in the provider. This is consistent with the literature on expectancy effects, which suggest that belief in treatment effectiveness plays a crucial role (Boettcher et al., 2013; Pontén et al., 2023). Therefore, it is important to examine factors that shape expectations about online treatments, which appear equally susceptible to expectancy effects as face-to-face treatments (Pontén et al., 2023). Misconceptions about therapy being inherently enjoyable rather than challenging also contributed to dropouts. Both early and midway dropouts found the intervention demanding and unpleasant. Setting realistic expectations about the nature of therapy, including its potential to be challenging and even temporarily worsen symptoms (Rozental et al., 2015), could help reduce early dropout rates.

### 4.2 Participant selection

The suitability of online treatment for different clients remains uncertain. Some participants left the intervention because their mental health issues were too severe, while completers sometimes attributed their success to having milder symptoms. Previous research suggests online treatments may be more effective for those with milder symptoms (Rozental et al., 2019), though this is not always the case (Karyotaki et al., 2018). Precise eligibility criteria are still unclear, but a thorough pre-assessment could enhance participant matching and intervention outcomes. Some completers preferred online care, while a third of dropouts sought other forms of care. Brief interventions might be better suited to students' schedules (Ciharova et al., 2023), suggesting that careful recruitment and information are crucial.

### 4.3 Interactive functionalities

Participants expressed a need for enhanced interactive features. Issues included overwhelming amounts of text and open-ended questions. To address these concerns, incorporating more closed questions and providing immediate feedback could improve user experience. While this approach was used during the screening phase, it was not applied during the intervention (Van der Heijde et al., 2015). Participants also felt that reminders were insufficiently frequent and easy to ignore. Increasing the frequency and variety of reminders, such as through mobile notifications, could be beneficial. Adding more engaging, game-like features might also enhance interaction and adherence (Fleming et al. 2017). Chatbot interfaces, positively reviewed by a large majority of nearly 8000 users in a recent study, could be explored as well (Malik et al., 2022).

### 4.4 Personalized care

A significant issue was the perceived lack of personalization. Some dropouts felt their issues were too severe or not adequately addressed by the intervention. Future trials could improve by offering more customizable options so that exercises are tailored to individual needs. Additionally, using more advanced algorithms instead of fixed templates to provide personalized feedback based on individual responses could increase relevance. Another approach could be to provide feedback on demand, which could better align with participants' needs.

### 4.5 Human guidance

Dropouts in this study often reported insufficient human interaction, while completers valued the sense of care and support. Yet, current research on iCBT shows that the addition of human-based guidance does not always lead to better results (Karyotaki et al., 2021; Lungu et al., 2022; Koelen et al., 2024). The optimal type and amount of feedback likely varies between individuals, which has some clear clinical implications (see remarks in 4.3 and 4.4). Research indicates that elements like “self-efficacy shaping” and empathetic communication are crucial for engagement and completion (Paxling et al., 2013). The challenge for future online treatments is to successfully integrate compassion and relational aspects into the therapeutic process (van Lotringen et al., 2023). There are other ways to include “guidance” outside of the client-therapist dyad. Several students mentioned that they missed the option of peer support, while others noted that external encouragement helped them persevere. It seems beneficial to advise participants to involve someone from their personal network, but this issue should be subjected to further study. Finally, advancements in AI offer potential to support clinicians in decision-making and feedback provision, potentially increasing adherence (Sadeh-Sharvit et al., 2023).

### 4.6 Strengths and limitations

This study provides valuable insights into the experiences of university students with iCBT interventions, offering essential information for improving future implementations in educational settings. As a core strength, it examined various stages of treatment completion (early drop-out, midway drop-out, and completion), assessing adherence as a multidimensional construct. Nonetheless, some limitations should be noted when interpreting the results. The relatively small sample sizes limit the generalizability of the findings to other student samples. The COVID-19 pandemic, which coincided with data collection, may have influenced dropout rates, as some participants cited pandemic-related changes as reasons for discontinuation. At the same time, psychological support during this period was predominantly offered online, and for some students, the online format may have also provided increased accessibility. Replication of this study outside the context of a pandemic would help to determine the extent to which our findings were shaped by the unique circumstances of COVID-19. The study combined data from human-guided and computer-guided intervention arms due to small sample sizes, which limits the ability to assess the impact of human guidance separately. Additionally, excluding PhD students from the sample may affect the applicability of findings to other university student populations. A further limitation of this study is the non-systematic sampling strategy. As we prioritized more recent participants, the findings may not fully capture the perspectives of all individuals who dropped out at different stages. Future research using more representative sampling could further validate and extend our findings. Finally, the study did not analyze the relationship between baseline symptom severity and dropout rates.

### 4.7 Conclusions

iCBT interventions provide an efficacious and accessible means to address the mental health needs of university students. However, high dropout rates may limit their impact. This study showed that both internal factors and external factors influenced dropout at different stages of treatment. Our findings underscore the importance of careful participant selection and managing expectations regarding the content and format of these interventions.

These insights enhance our understanding of adherence-related factors and offer a foundation for more targeted, fine-grained research on dropout. Future studies could explore ways to further personalize care through AI-driven interactive functionalities, as well as optimizing the type and level of individualized guidance to better meet participants' needs.

## Declaration of Generative AI and AI-assisted technologies in the writing process

During the preparation of this work the author(s) used ChatGPT to improve the readability of the manuscript. After using this tool/service, the author(s) reviewed and edited the content as needed and take(s) full responsibility for the content of the published article.

## Declaration of competing interest

The authors declare that they have no known competing financial interests or personal relationships that could have appeared to influence the work reported in this paper.

## Appendix A Supplementary data

Appendix A Interview protocol. Image 1
