# Supplementary material for: Dropout and completion in iCBT for university students: Insights from a thematic analysis
Source: Internet Interv. 2025 May 14;40:100831. doi: 10.1016/j.invent.2025.100831 (PMC12148420; doi:10.1016/j.invent.2025.100831)
Supplement: Supplementary file 1 — Appendix A Interview protocol. [file mmc1.docx]

Appendix A Interview Protocol

Introduction

Good morning/good afternoon, this is [*interviewer’s name*] from [*project*] at [*university*]. Am I speaking with [*interviewee’s name*]? Great. I am contacting you about your participation in [*project*]. Is this a convenient time for you?

🡪 Yes: Continue.

🡪 No: Reschedule.

As you may know, we are investigating the usefulness of this online self-help training. We want the online health support to match the needs and wishes of students as best as possible, and we are looking for ways to improve it. Can we ask you some questions regarding your experience with [*project*] and the online training?

🡪 Yes: Continue.
🡪 No: End conversation.

Great! Before we start, I would like you to know that you can answer these questions honestly and openly. This information is solely for us to get a better insight into your experience and for future improvements. The information you share will be processed anonymously. For our research to be accurate, this interview will be recorded – do you agree to this?

🡪 Yes: Great, thank you. We will never link your personal details to your answers.
🡪 No: We will not record this conversation. [*stop and delete recording; end interview*]

A few months ago, you had an intake interview by phone, after which you filled in the baseline questionnaire. Next you received an email inviting you to participate in the online self-help training. So far, it looks like you have completed [*n*] session(s).

Questions: Early dropout

1. What is the main reason you chose to participate in [*project*]?
2. What is the main reason you have not started or completed the first session?
3. Are there any other reasons you have not started or completed the first session?
4. Hypothetically, if you were sent the invitation to participate in the training today, would you start? Why, or why not?
5. What were your expectations for this training? Did these expectations from the actual programme?
6. What was your first impression of the training?
7. What would have been necessary for you to complete the first session?
8. In general, what is your opinion of [*project*]?
9. What tips do you have to improve [*project*]?
10. Is there anything we haven’t discussed yet that you would like to share about the intervention?

Questions: Midway dropout

1. What is the main reason you chose to participate in [*project*]?
2. What was your first impression of the training?
3. What was your experience of the training?
4. What were your expectations for this training? Did these expectations differ from the actual programme?
5. What is the main reason you did not continue with the training?
6. In your opinion, was there anything missing from the training? If so, what was it?
7. What would have been needed for you to finish all sessions?
8. In general, what is your opinion of [*project*]?
9. (TG only) What is your opinion of the guidance you received?
10. (TG only) How can we improve this guidance?
11. Overall, what tips do you have to improve [*project*]?
12. Is there anything we haven’t discussed yet that you would like to share about the training?

Questions: Completers

1. What is the main reason you chose to participate in [*project*]?
2. What was your first impression of the training?

3. What was your experience of the training?
4. What were your expectations for this training? Did these expectations differ from the actual programme?
5. In your opinion, was there anything missing from the training? If so, what was it?
6. What did you think was most helpful?
7. What did you think was least helpful?
8. (TG only) What is your opinion of the guidance you received?
9. (TG only) How can we improve this guidance?
10. Overall, what tips do you have to improve [*project*]?
11. Is there anything we haven’t discussed yet that you would like to share about the training?

Conclusion

These were all the questions we have for you. Do you have any questions for us?

I want to thank you for sharing your experience with us. Your input will help us improve this programme in the future.
